# Do 360-degree Feedback Survey Results Relate to Patient Satisfaction Measures?

**DOI:** 10.1007/s11999-014-3981-3

**Published:** 2014-10-07

**Authors:** Michiel G. J. S. Hageman, David C. Ring, Paul J. Gregory, Harry E. Rubash, Larry Harmon

**Affiliations:** 1 Massachusetts General Hospital, Boston, MA USA; 2 PULSE 360 Program/Physicians Development Program, 2000 S Dixie Highway, Suite 103, Miami, FL 33133 USA

## Abstract

**Background:**

There is evidence that feedback from 360-degree surveys—combined with coaching—can improve physician team performance and quality of patient care. The Physicians Universal Leadership-Teamwork Skills Education (PULSE) 360 is one such survey tool that is used to assess work colleagues’ and coworkers’ perceptions of a physician’s leadership, teamwork, and clinical practice style. The Clinician & Group-Consumer Assessment of Healthcare Providers and System (CG-CAHPS), developed by the US Department of Health and Human Services to serve as the benchmark for quality health care, is a survey tool for patients to provide feedback that is based on their recent experiences with staff and clinicians and soon will be tied to Medicare-based compensation of participating physicians. Prior research has indicated that patients and coworkers often agree in their assessment of physicians’ behavioral patterns. The goal of the current study was to determine whether 360-degree, also called multisource, feedback provided by coworkers could predict patient satisfaction/experience ratings. A significant relationship between these two forms of feedback could enable physicians to take a more proactive approach to reinforce their strengths and identify any improvement opportunities in their patient interactions by reviewing feedback from team members. An automated 360-degree software process may be a faster, simpler, and less resource-intensive approach than telephoning and interviewing patients for survey responses, and it potentially could facilitate a more rapid credentialing or quality improvement process leading to greater fiscal and professional development gains for physicians.

**Questions/purposes:**

Our primary research question was to determine if PULSE 360 coworkers’ ratings correlate with CG-CAHPS patients’ ratings of overall satisfaction, recommendation of the physician, surgeon respect, and clarity of the surgeon’s explanation. Our secondary research questions were to determine whether CG-CAHPS scores correlate with additional composite scores from the Quality PULSE 360 (eg, insight impact score, focus concerns score, leadership-teamwork index score, etc).

**Methods:**

We retrospectively analyzed existing quality improvement data from CG-CAHPS patient surveys as well as from a department quality improvement initiative using 360-degree survey feedback questionnaires (Quality PULSE 360 with coworkers). Bivariate analyses were conducted to identify significant relationships for inclusion of research variables in multivariate linear analyses (eg, stepwise regression to determine the best fitting predictive model for CG-CAHPS ratings). In all higher order analyses, CG-CAHPS ratings were treated as the dependent variables, whereas PULSE 360 scores served as independent variables. This approach led to the identification of the most predictive linear model for each CG-CAHPS’ performance rating (eg, [1] overall satisfaction; [2] recommendation of the physician; [3] surgeon respect; and [4] clarity of the surgeon’s explanation) regressed on all PULSE scores with which there was a significant bivariate relationship. Backward stepwise regression was then used to remove unnecessary predictors from the linear model based on changes in the variance explained by the model with or without inclusion of the predictor.

**Results:**

The Quality PULSE 360 insight impact score correlated with patient satisfaction (0.50, p = 0.01), patient recommendation (0.58, p = 0.002), patient rating of surgeon respect (0.74, p < 0.001), and patient impression of clarity of the physician explanation (0.69, p < 0.001). Additionally, leadership-teamwork index also correlated with patient rating of surgeon respect (0.46, p = 0.019) and patient impression of clarity of the surgeon’s explanation (0.39, p = 0.05). Multivariate analyses supported retention of insight impact as a predictor of patient overall satisfaction, patient recommendation of the surgeon, and patient rating of surgeon respect. Both insight impact and leadership-teamwork index were retained as predictors of patient impression of explanation. Several other PULSE 360 variables were correlated with CG-CAHPS ratings, but none were retained in the linear models post stepwise regression.

**Conclusions:**

The relationship between Quality PULSE 360 feedback scores and measures of patient satisfaction reaffirm that feedback from work team members may provide helpful information into how patients may be perceiving their physicians’ behavior and vice versa. Furthermore, the findings provide tentative support for the use of team-based feedback to improve the quality of relationships with both coworkers and patients. The 360-degree survey process may offer an effective tool for physicians to obtain feedback about behavior that could directly impact practice reimbursement and reputation or potentially be used for bonuses to incentivize better team professionalism and patient satisfaction, ie, “pay-for-professionalism.” Further research is needed to expand on this line of inquiry, determine which interventions can improve 360-degree and patient satisfaction scores, and explain the shared variance in physician performance that is captured in the perceptions of patients and coworkers.

**Electronic supplementary material:**

The online version of this article (doi:10.1007/s11999-014-3981-3) contains supplementary material, which is available to authorized users.

## Introduction

Anonymous survey feedback from physicians and nursing/staff team members (360-degree feedback) and the individual summary reports based on that feedback are increasingly recommended and used for quality improvement for medical students, residents, and physicians [7, 10, 11, 13–15, 18, 25]. Physician feedback using 360-degree surveys along with goal-setting, coaching, and/or other developmental interventions have shown improvement in all six Accreditation Council for Graduate Medical Education (ACGME) Core Competency-related behavioral scales for residents [11] and interpersonal skills for “disruptive” physicians [13]. One recently developed 360-degree feedback survey, the Quality PULSE (Physicians Universal Leadership Skills Education) 360 survey (see Appendix 1 for more information on the PULSE 360 [Supplemental materials are available with the online version of CORR^®^.]), provides screening feedback regarding the surgeon’s six core competencies endorsed by the ACGME, American Board of Medical Specialties, and The Joint Commission while emphasizing interpersonal and communication skills, professionalism, and safety culture. The Clinician & Group-Consumer Assessment of Healthcare Providers and System (CG-CAHPS) survey measures the patient’s perception of his or her visit [1, 2] and includes 28 questions, of which five are used to assess the access to care, six to assess communication, and two to assess courteous/helpful staff (see Appendix 2 for more information on the CG-CAHPS [Supplemental materials are available with the online version of CORR^®^.]).

Routine 360-degree feedback is recommended as a cost-effective tool to prevent or reduce disruptive behavior and improve physician emotional intelligence, leadership, teamwork, clinical, and financial and organizational outcomes such as patient satisfaction [7, 13–15, 18]. Physician emotional intelligence has been associated with improved nurse-physician relationships [5, 6, 12, 19–22] as well as higher patient satisfaction [3–5, 11, 26, 27]. Several important components comprise the mechanism of how emotional intelligence impacts work life: self-awareness, understanding others, building relationships, listening effectively, communicating convincingly, avoiding or resolving conflicts, and positively guiding and motivating team members [8]. These emotional and social skills may be the behavioral advantage more emotionally intelligent physicians hold over their less emotionally intelligent peers. Prior research has indicated that patients and coworkers often agree in their assessment of physicians’ behavioral patterns [7, 14, 15, 18, 25]. However, relatively little research has examined how coworkers’ perceptions of a physician’s workplace behavioral performance translate to patient-based performance. Patient satisfaction measures are increasingly used for benchmarking clinical quality [3, 4, 16, 17]; therefore, being able to predict patient satisfaction is becoming even more valuable to hospitals and providers than ever before. One of the most common and popular patient satisfaction measures is the CG-CAHPS [1, 2] patient feedback survey tool developed by the US Department of Health and Human Services, which has recently become a benchmark tied to Medicare-based compensation of participating physicians.

The goal of the current study was to determine whether multisource feedback stemming from coworkers (Quality PULSE 360) could be used to predict patient satisfaction/experience ratings (CG-CAHPS). A significant relationship between these two forms of feedback would allow physicians/hospitals to take a more proactive approach to forecasting their patient performance by examining performance with coworkers. Gathering feedback from coworkers is a much simpler and less resource-intensive process than gathering feedback from patients; therefore, a process of quality improvement driven by coworker-based feedback could lead to greater fiscal and professional development gains for physicians and hospitals alike.

Specifically, we investigated the following research questions: (1) Do CG-CAHPS’ patient satisfaction scores for physicians positively correlate with the Quality PULSE 360 insight impact score? (2) Which PULSE 360 domains correlate with CG-CAHPS patients’ ratings for overall satisfaction, recommendation of the physician, surgeon respect, and impression of the surgeon’s explanation? Our secondary research questions were to determine whether CG-CAHPS scores correlate with additional composite scores from the PULSE 360 (including focus concerns, leadership teamwork index, and core competency composite score). Additionally, we sought to determine if predictive models could be determined for CG-CAHPS ratings based on significant relationships with PULSE 360 ratings.

## Materials and Methods

### Study Design and Setting

After receiving approval from our institutional research board, we conducted a retrospective study using existing CG-CAHPS survey data collected as part of routine hospital operations and data from the department’s 360-degree survey feedback-based quality improvement initiative (Quality PULSE 360).

### Participants/Study Subjects

The mean age of the 26 orthopaedic surgeons who participated in the Quality PULSE 360 initiative was 50 years (SD, 10; range, 35–71 years) and 25 of 26 (96%) were men (Table 1). Baseline (first time participating) PULSE 360 survey data were collected for all 26 surgeons between 2011 and 2013 by inviting the physician peers and clinical/administrative healthcare team members with whom each surgeon works most often, selected by both the surgeon as well as by the chief, to provide feedback about their perceptions of the leadership, teamwork, and clinical practice style of that surgeon. The 26 surgeons represent all the physicians who were full-time attendings within the department. The CG-CAHPS satisfaction survey data were obtained by phone survey for outpatient visits from February 2008 through June 2013.

**Table 1:** Summary statistics (n = 26 surgeons)

Variable	Mean	SD	Minimum	Maximum
Age (years)	50	10	35	71
CAHPS				
Rating physician’s explanation, n = 7449 patients	5	0.2	4.9	5.7
Rating physician’s respect, n = 7318	5	0.2	5.0	5.8
Rating satisfaction about treating physician, n = 8064 patients	9	0.4	7.8	9.7
Recommending treating physician, n = 8021 patients	3	0.1	3.4	4.0
PULSE data				
Motivating behavior	4	0.3	3.8	4.8
Discouraging behavior	1	0.4	1.0	2.4
Physician core competency item	4	0.4	3.3	4.9
Insight impact*	4	0.4	3.2	4.8
Focus concerns^†^	1	0.3	1.0	2.2
Burnout concerns^ǂ^	1	0.4	1.0	2.5
Leadership teamwork index	75	17	36	93
Technical competency index	90	8.5	60	98
Nontechnical competency index	92	4.8	77	97
Surgical-procedural skills	14	0.9	11	15
Patient care	8	1.3	5.4	10
Medical knowledge	9	0.6	6.8	10
Practice-based learning and improvement	9	0.4	8.3	10
Systems-based practice	4	0.2	4.2	5.0
Professionalism	14	0.6	13	15
Interpersonal and communications skills	9	0.6	7.2	10

* Understands how his or her behavior impacts others; ^†^perceived distraction, disorganization, confusion, or absent-mindedness; ^ǂ^perceived tiredness or being overworked; CAHPS = Consumer Assessment of Healthcare Providers and System; PULSE = Physicians Universal Leadership-Teamwork Skills Education.

### Variables, Outcome Measures, Data Sources, and Biases

The CG-CAHPS survey is a program of the US Agency for Healthcare Research and Quality and commonly used to assess the patient’s experience and perception of care in the ambulatory medical office setting [1, 2]; it is a 28-item survey that provides patient feedback on access to care, doctor communication, courteous/helpful staff, overall doctor rating, and likelihood of recommending doctor rating. The CG-CAHPS patient satisfaction question was completed by 8064 patients, the willingness to recommend the doctor to family and friends by 8021 patients, the doctor’s explanation by 7449 patients, and how much the doctor showed respect by 7318 patients. The inconsistency in patient rating counts for each item was the result of missing data or incomplete surveys.

The Quality PULSE 360 survey consists of 44 questions scored on a Likert-type extent scale (see Appendix 1 for more information on the PULSE 360). The PULSE survey has been administered to over 5000 participants throughout the United States and Canada with over 100,000 completed surveys, or an average of 20 raters per survey. PULSE 360 survey ratings were organized into 10 composite scores for analyses: (1) motivating behaviors; (2) discouraging behaviors; (3) core competency behaviors; (4) insight impact score (understands how his or her behavior impacts others); (5) focus concerns score (ie, perceived distraction, disorganization, confusion, or absent-mindedness); (6) burnout concerns score (ie, perceived tiredness or being overworked); (7) leadership/teamwork index score (ie, proprietary index calculated using motivating and discouraging behaviors together); (8) technical competency index (ie, all practice items specific to clinical treatment under the subcategories of: patient care, medical knowledge, practice-based learning and improvement, and systems-based practice); (9) nontechnical competency index (ie, all practice items related to the subcategories of: professionalism, and interpersonal and communication skills); and (10) surgical-procedural skills (ie, all surgical specialty-specific items). The mean number of PULSE raters per surgeon was 22 (SD, 11).

Colleagues, peers, managers, nurses, technicians, and trainees anonymously completed the 360-degree Quality PULSE 360 providing their perceptions of the surgeon’s behavior based on their last 12 months of interaction with that surgeon. All CG-CAHPS and PULSE 360 data were coded so that the surgeon could not be identified and only the principal investigator had access to the key. The key was stored on the principal investigator’s locked and encrypted computer.

### Statistical Analysis, Study Size

The primary independent variable was the Quality PULSE 360 insight impact score. The secondary independent variables were all other composite PULSE 360 scores. The primary dependent variable was patient satisfaction based on the average CG-CAHPS overall rating of the physician. The secondary dependent variables were the willingness to recommend the doctor to family and friends, the rated ease of understanding the doctor’s explanations, and the extent to which the doctor showed respect.

The relationship between continuous variables was tested using the Spearman rho test. The Pearson chi-square test was used to determine the differences between categorical variables unless the minimum expected cell frequency was less than five, in which case the Fisher’s exact test was used instead. Wilcoxon signed rank tests were performed to determine the differences between continuous and dichotomous variables. Multivariate linear models were developed based on the results to the initial bivariate correlations. Specifically, Quality PULSE 360 scores were included in the model for each CG-CAHPS outcome variable if a significant bivariate relationship existed. The model was then reduced by backward stepwise regression to determine the model with the most variance explained using the fewest explanatory variables.

## Results

The final regression model for patient satisfaction included insight impact alone and accounted for 12% of the variability (p = 0.047) (Table 2). The final regression model for patient recommendation of the treating physician included PULSE 360 insight impact alone and accounted for 17% of the variability (p = 0.023). The final regression model for surgeon respectful behavior included PULSE insight impact alone and accounted for 45% of the variability (p < 0.001). The final regression model for patient impression of the surgeon’s explanation included PULSE 360 insight impact and leadership teamwork index and accounted for 48% of the variability (p < 0.001).

**Table 2:** Multivariable analysis

Variable	Coefficient	p value	95% confidence interval		Adjusted R^2^
			Low	High	
Patients satisfaction					
Insight impact	0.38	0.047	0.01	0.76	0.12
Patient’s recommendation of the treating physician					
Insight impact	0.15	0.023	0.02	0.27	0.17
Patient’s rating of surgeon respect					
Insight impact	0.29	<0.001	0.16	0.42	0.45
Patient’s impression of the surgeon’s explanation					
Leadership teamwork index	−0.01	0.031	−0.02	0.00	0.48
Insight impact	0.62	<0.001	0.31	0.94	

Before multivariate analysis, initial bivariate analysis explored PULSE 360 variables with CG-CAHPS ratings (Table 3). As expected, we found that insight impact correlated with patient satisfaction (0.50, p = 0.010), patient recommendation (0.58, p = 0.0020), patient rating of surgeon respect (0.74, p < 0.001), and patient impression of explanation (0.69, p < 0.001). Additionally, leadership-teamwork index also correlated with patient rating of surgeon respect (0.46, p < 0.019) and patient impression of explanation (0.39, p = 0.05; Table 3). Focus concerns (ie, distracted, disorganized, etc) were negatively correlated with patient satisfaction (−0.55, p = 0.0036), patient recommendation (−0.64, p < 0.001), patient rating of surgeon respect (−0.60, p = 0.0013), and patient impression of explanation (−0.57, p = 0.0026). Nontechnical competency index was correlated with patient recommendation (0.39, p = 0.05) and patient impression of explanation (0.47, p = 0.016). Lastly, interpersonal and communication skills were correlated with patient recommendation (0.50, p = 0.009), patient rating of surgeon respect (0.41, p = 0.036), and patient impression of explanation (0.56, p = 0.0027).

**Table 3:** Bivariable analysis

	CAPHS							
	Patient satisfaction		Patient’s recommendation of the treating physician		Patient rating of surgeon respect		Patient’s impression of the surgeon’s explanation	
	Spearman r	p value	Spearman r	p value	Spearman r	p value	Spearman r	p value
Age	−0.29	0.15	−0.30	0.14	−0.07	0.72	−0.11	0.58
PULSE								
Motivating behavior	0.31	0.12	0.35	0.08	**0.49**	**0.011**	**0.42**	**0.034**
Discouraging behavior	−0.19	0.34	−0.25	0.22	−**0.47**	**0.015**	−**0.40**	**0.041**
Physician core competency item	0.15	0.47	0.19	0.35	0.25	0.22	0.36	0.068
Insight impact*	**0.50**	**0.010**	**0.58**	**0.0020**	**0.74**	**< 0.001**	**0.69**	**< 0.001**
Focus concerns^†^	−**0.55**	**0.0036**	−**0.64**	**< 0.001**	−**0.60**	**0.0013**	−**0.57**	**0.0026**
Burnout concerns^ǂ^	−0.12	0.68	−0.29	0.32	−0.09	0.77	−0.33	0.249
Leadership teamwork index	0.24	0.24	0.30	0.14	**0.46**	**0.019**	0.39	0.050
Technical competency index	0.12	0.58	0.12	0.55	0.23	0.25	0.30	0.14
Nontechnical competency index	0.31	0.12	0.39	0.05	0.29	0.15	**0.47**	**0.016**
Surgical-procedural skills	−0.04	0.85	−0.04	0.86	−0.10	0.64	0.05	0.80
Patient care	−0.08	0.69	−0.03	0.88	0.03	0.90	0.14	0.51
Medical knowledge	0.15	0.46	0.16	0.45	0.12	0.58	0.22	0.27
Practice-based learning and improvement	0.16	0.42	0.18	0.39	0.084	0.68	0.18	0.37
Systems-based practice	0.11	0.59	0.22	0.28	−0.097	0.64	0.02	0.94
Professionalism	0.29	0.20	0.34	0.14	0.15	0.51	0.31	0.18
Interpersonal and communications skills	0.36	0.067	**0.50**	**0.009**	**0.41**	**0.036**	**0.56**	**0.0027**

Bold values are significant; * understands how his or her behavior impacts; ^†^perceived distraction, disorganization, confusion, or absent-mindedness; ^ǂ^perceived tiredness or being overworked; CAHPS = Consumer Assessment of Healthcare Providers and System; PULSE = Physicians Universal Leadership-Teamwork Skills Education.

## Discussion

There is a growing trend toward using 360-degree feedback as a tool to help improve professionalism, interpersonal and communication skills, and quality of care [7, 13–15, 18, 25]. Additionally, patient satisfaction measures are increasingly being used as a benchmark for physician clinical quality [3, 4, 9, 19, 21, 27]. The goal of the current project was to determine whether data from a 360-degree feedback survey correlate with, and could potentially be used to predict, patient satisfaction ratings. As mentioned earlier, existing research has demonstrated that patients and coworkers often show agreement in their ratings of a physician’s behavior [7, 14, 15, 18, 25]. However, very little research has explored the perceptual overlap of patients and coworkers when it comes to clinical performance of physicians. As such, we wanted to examine retrospective 360-degree feedback from coworkers and analyze the scores against patient satisfaction data to determine if a predictive relationship exists between coworker feedback and patient feedback that could offer physicians and hospitals a simple methodology to forecast and improve clinical quality [3, 4, 9, 16–18, 20, 21]. The findings of the current study provide preliminary support for the assertion that 360-degree feedback from coworkers may be predictive of various measures of patient satisfaction/experience. Specifically, a physician’s ability to understand how his or her behavior impacts coworkers is predictive of CG-CAHPS ratings related to overall satisfaction, recommendation of the physician, patient rating of physician respect, and, along with their overall leadership/teamwork skill, is predictive of patient’s impression of explanation of their treatment/visit.

However, the results of this study should be interpreted in light of its shortcomings. One limitation is the limited number of surgeons (N = 26), and so some of our no-difference findings may in fact be found to be important in future larger studies. Likewise, the 95% confidence intervals and the strength of the prediction models should be interpreted with caution. Furthermore, the strong correlation of PULSE 360 seemed to create collinearity so that the insight impact score was the only significant explanatory variable in several final models. Finally, these data might only apply to this particular 360-degree survey, sample of surgeons, specialty, hospital, or geographic region. However, this is unlikely given the pattern of significant observed relationships being based on a relatively small sample that was representative of all full-time orthopaedic physicians in that department.

The relationship between PULSE insight impact and CG-CAHPS overall rating of physician, recommendation of physician, physician respect, and physician clarity supports extant research [3, 4, 9, 16, 21, 26, 27] and prior research demonstrating emotional intelligence as a key predictor of a physician success in both work team and patient relationships. For instance, one study found that patient satisfaction correlated with teamwork, safety climate, and stress recognition, but not with Surgical Care Improvement Program metrics (eg, antibiotic prophylaxis, hair removal, etc) [16]. A review of patient experience literature [4] found that patient satisfaction correlated most highly with a physician’s interpersonal caring behaviors such as good communication skills, empathy, and sensitivity to patient needs. Researchers performed a telephone survey of 4985 patients with one of four conditions treated at one of 126 hospitals in Taiwan, and they found that patient rating of interpersonal skills was a stronger determinant of patient satisfaction than patient rating of competence [3]. On the other hand, other researchers interviewed 10,250 patients in 77 Japanese hospitals and found that ratings of physician competence were significantly associated with overall satisfaction [24]. In a meta-analysis of 221 studies of patient satisfaction with medical care performed, researchers identified humaneness and technical quality as the most important factors influencing satisfaction [9]. A study of family practice physicians found that patient trust correlated more strongly with comforting and caring, technical competency, and communication style than with gentleness, looking in the eye, discussion of options, and treatment as an equal [23].

The relationship between Quality PULSE 360 focus concerns and CG-CAHPS patient’s overall rating/recommendation of physician reinforces the importance of patient perception of their physician’s competence [5, 9, 16]. Although team members’ perceptions of competence were not significantly related to patients’ overall rating/recommendation of physician, this may be because team members’ understanding and evaluation of a physician’s competence occurs at a more sophisticated level than patients’ perceptions. Work team members are able to evaluate clinical competence based on their own experience and training [7, 14, 15, 25], whereas patients may need to rely on more basic indicators such as whether their physician seems distracted, disorganized, etc [9, 24, 27]. PULSE 360 focus concerns may capture work team members’ perception of some of those more basic competence indicators similar to how a patient will evaluate the competence of their treating physician.

The finding in this study that patient recommendation of the physician did not correlate with work team members’ impressions of technical competency is inconsistent with prior research that identified technical skills [9] as the strongest predictor of patients’ recommendation. However, the aforementioned divergent relationship and ability of patients and work team members to evaluate physician behavior may explain the lack of significant relationships between the CG-CAHPS patient overall rating/recommendation of the physician and the additional Quality PULSE 360 scores (leadership-teamwork index, competency composite score, etc). Because patients have relatively limited interactions with their physicians compared with work team members, a physician’s ability to interact/communicate in a professional manner with work team members may have little bearing on patient overall rating/recommendation of that physician. This observation is further supported when we consider that patients also have a limited ability to observe their physicians interacting with their work team members and are likely more interested in and attentive to how their physician interacts/communicates with them.

Conversely, the significant findings that link PULSE 360 scores related to interpersonal/communication skills and patient’s perceptions of physician respect and clarity demonstrate that there is likely a conceptual overlap in ability to evaluate that behavior for both patients and work team members [7, 9, 17, 18, 21]. If a physician is respectful to work team members, then it follows that the physician will likely be respectful with patients. Similarly, if a physician communicates effectively with work team members, it follows that the physician is likely to communicate effectively with patients. Based on these findings, it appears that the ACGME core competency of interpersonal and communication skills, compared with the other five core competencies, is the most readily observable/assessable proficiency by both patients and work team members. Future research will need to investigate this topic further as it reaffirms the value of training/ongoing development of a physician’s “soft skills” [5].

Future research will need to address the shortcomings and replicate the parameters of this study to provide a more comprehensive evaluation of the relationship between team members’ perceptions of a physician’s quality and patients’ perceptions of a physician’s quality. More meaningful measures of physician behavior and interventions are needed that can help physicians improve their individual performance and contribution to care teams [27]. Our data suggest that anonymous 360-degree feedback from healthcare team members provides information that correlates with the patient experience [9, 16, 17, 26, 27]. The Quality PULSE 360 may be one such survey tool that can offer physicians valuable feedback that if leveraged may impact both their work team and patient relationships in significantly positive ways. Future research should confirm these findings and further determine if additional interventions such as standardized debriefings and/or coaching based on 360-degree data can lead to improvements in both team member and patient-based quality measures.

## Electronic supplementary material

Below is the link to the electronic supplementary material.

Supplementary material 1 (DOCX 13 kb)

Supplementary material 2 (DOCX 13 kb)
