# Supplementary material for: Do 360-degree Feedback Survey Results Relate to Patient Satisfaction Measures?
Source: Clin Orthop Relat Res. 2014 Oct 7;473(5):1590–7. doi: 10.1007/s11999-014-3981-3 (PMC4385380; doi:10.1007/s11999-014-3981-3)
Supplement: Supplementary file 1 — Supplementary material 1 (DOCX 13 kb) [file 11999_2014_3981_MOESM1_ESM.docx]

**Appendix 1.** PULSE 360 Survey Information
The Quality PULSE 360 survey consists of 44 questions scored on a Likert-type extent scale divided into six main behavioral dimensions: (1) motivating behaviors; (2) discouraging behaviors; (3) insight impact; (4) physician core competencies; (5) focus concerns; and (6) burnout concerns. Motivating behaviors include behaviors like treating team members with respect, remaining approachable when stressed, communicating clear requests, etc. Discouraging behaviors include talking down to team members, blaming others for one’s mistakes, avoiding responsibilities, etc. For all questions, raters were asked to describe their observations/experiences involving the participant using a 5-point Likert-type extent with 1 reflecting “not at all” and 5 reflecting “to a very great extent.”

PULSE = Physicians Universal Leadership-Teamwork Skills Education.
