# Supplementary material for: Do 360-degree Feedback Survey Results Relate to Patient Satisfaction Measures?
Source: Clin Orthop Relat Res. 2014 Oct 7;473(5):1590–7. doi: 10.1007/s11999-014-3981-3 (PMC4385380; doi:10.1007/s11999-014-3981-3)
Supplement: Supplementary file 2 — Supplementary material 2 (DOCX 13 kb) [file 11999_2014_3981_MOESM2_ESM.docx]

**Appendix 2.** CG-CAHPS Survey Information
The CG-CAHPS measures the patient’s perception of his or her visit [21]. The survey includes 28 questions, of which five are used to assess the access to care, six to assess communication, and two to assess courteous/helpful staff. The survey also includes four questions that ask respondents (1) if the doctor explained things in a way that was easy to understand; (2) if the doctor showed respect; (3) if they would recommend this doctor to family and friends; and (4) to provide their doctor with an overall rating. Questions (1) about “explaining clearly” and (2) “respecting” were answered using a 6-point response scale. Question (3) about recommending the doctor was rated on a 4-point scale. The Doctor Overall Rating asked the patient to rate the doctor on an 11-point Likert scale, from 0 to 10, with 0 reflecting the worst doctor possible and 10 representing the best doctor possible. For questions (1) and (2), patients were asked to provide ratings based on the prior 12 months.

CG-CAHPS = Clinician & Group-Consumer Assessment of Healthcare Providers and System.
